# Testing the Efficacy of a Web-Based Intervention for Loss and Bereavement in Later Life (AgE-health Study): Results From a Randomized Controlled Trial

**DOI:** 10.2196/68662

**Published:** 2025-09-09

**Authors:** Franziska D Welzel, Alexander Pabst, Janine Stein, Franziska Bleck, Steffi G Riedel-Heller, Margrit Löbner

**Affiliations:** 1 Institute of Social Medicine, Occupational Health and Public Health (ISAP) Medical Faculty, University of Leipzig Leipzig Germany

**Keywords:** grief, late life, RCT, randomized controlled trial, eHealth, intervention

## Abstract

**Background:**

The loss of a loved one is a common yet stressful event in later life. Internet- and mobile-based interventions have been proposed as an effective treatment approach for individuals with prolonged grief.

**Objective:**

The AgE-health study aimed to investigate the efficacy of an eHealth intervention, trauer@ktiv, in reducing prolonged grief symptoms in a sample of older adults. The trauer@ktiv intervention is an unguided, web-based self-management intervention based on cognitive behavioral therapy principles, addressing grief in later life.

**Methods:**

The AgE-health study was designed as a randomized controlled trial with an active control group (CG). Recruitment and data assessment took place between October 2020 and September 2022. A sample of 177 older adults (aged ≥60 years) with prolonged grief symptoms was randomly assigned to the intervention group (IG; access to the trauer@ktiv eHealth intervention) or the active CG (access to an information brochure on loss and grief). Participants were interviewed at 2 time points, before and after accessing the intervention, via postal questionnaires (baseline and follow-up 4 months after the intervention). The primary outcome was the reduction in prolonged grief symptoms assessed with the Prolonged Grief-13 scale. Adjusted mixed-effects regression models were used to test for changes in primary and secondary outcomes (eg, depression and self-efficacy) as part of an intention-to-treat (ITT) analysis. The study procedure was described in detail in the study protocol.

**Results:**

From baseline to 4 months after the intervention, 7 participants were lost to follow-up. Final analyses included a sample of 170 individuals (IG: n=81, 47.6%; CG: n=89, 52.4%). The study population had an average age of 67.3 (SD 6.4; range 60-95) years and consisted predominantly of female participants (129/170, 75.9%). While there was a pre-post-reduction in persistent grief symptoms, the ITT analysis showed no significant differences between IG and CG at follow-up (Prolonged Grief-13 scale, average marginal effect of 0.56, 95% CI –2.30 to 3.44, *P*=.70). Similarly, the ITT analysis showed no significant treatment effects for any of the secondary outcomes at follow-up. The trauer@ktiv intervention was rated high on satisfaction and usability. More than half of the IG (49/81, 61%) completed 5 or more of the 8 modules of the eHealth intervention.

**Conclusions:**

The tested eHealth intervention, trauer@ktiv, showed no superiority compared to an active CG in reducing prolonged grief symptoms in a sample of older bereaved individuals. Therefore, trauer@ktiv is not suitable as a stand-alone treatment. However, as the ease of use and satisfaction with the application were high, there may be potential for the intervention within a stepped and collaborative treatment approach.

**Trial Registration:**

German Clinical Trials Register DRKS00020595; https://drks.de/search/de/trial/DRKS00020595

**International Registered Report Identifier (IRRID):**

RR2-10.1016/j.invent.2021.100451

## Introduction

### Background

Persistent grief reactions accompanied by functional impairments (eg, problems connecting with other people) have been included as a new disorder under the term prolonged grief disorder (PGD) in the text revision of the *DSM-5* (*Diagnostic and Statistical Manual of Mental Disorders* [Fifth Edition]) [1], as well as the current revision of the *ICD-11* (*International Statistical Classification of Diseases and Related Health Problems, 11th Revision*) [2]. According to the ICD-11, PGD is understood as a persistent and pervasive grief response lasting for an unusually long period (at least 6 months) and exceeding the expected social norms for the individual’s cultural context. The prolonged grief response is characterized by the preoccupation or the longing for the deceased and is accompanied by intense painful or negative emotions, such as guilt, anger, difficulty accepting the death, or emotional numbness. The PGD diagnosis demands functional impairments in personal, occupational, or other important areas of the individual’s life [2].

In recent years, several epidemiological studies have investigated the prevalence of PGD in the general population. A cross-national analysis reported PGD prevalence rates ranging from 2% (Israel) to 35.5% (China) with an estimated overall prevalence of 13% across countries [3]. In Germany, the prevalence of PGD has been reported to be 3.7% in the general population with a conditional prevalence of 6.7% among bereaved individuals [4]. A recent review investigating the prevalence of PGD in older adults found large variations among the included studies, ranging from 3.4% to 26.2% in epidemiological studies and from 6% to 48.7% in nonepidemiological studies [5].

Death and the loss of loved ones, such as spouses, family members, or friends, is a particularly common occurrence in later life and requires a great deal of psychological adjustment on the part of the bereaved. Previous research has investigated a range of bereavement-related contextual variables, including the circumstances surrounding the death, the quality of the relationship with the deceased, and the availability of social support, to understand their impact on postloss psychopathology and the development of PGD. Several studies indicate that violent or unexpected losses are associated with more pronounced grief responses and an increased need for professional help [6,7]. It has been suggested that intense helplessness and dissociation at the time of death disrupt the encoding and the consolidation of loss-related memories, thereby prolonging acute grief reactions [6]. Furthermore, kinship ties and the perceived closeness to the deceased may influence postloss grief reactions. The loss of a child, in particular, is regarded as one of the most painful forms of bereavement [8]. While such a loss can be traumatic across all age groups, older adults may exhibit more complex grief responses due to age-related vulnerabilities. A recent review indicated that bereaved older parents are at heightened risk for death-related anxiety, social isolation, and cognitive decline [9]. In contrast, social support has the potential to strengthen the resilience of bereaved individuals as it has been shown to be positively associated with coping capacities [10]. In particular, emotional support may play a critical role in the context of traumatic loss [11].

Research has shown that prolonged grief can contribute substantially to the development of mental illnesses. PGD is often accompanied by anxiety, depression, somatization, or traumatic stress disorders, and it has been shown to predict an increase in substance abuse [12,13]. In addition, a recent review reported that bereavement in later life is linked to sleep issues, a higher use of medical services, and weight loss [5]. Furthermore, it has been suggested that PGD in later life may act as a possible risk factor for cognitive decline in older adults [14].

Despite these negative health impacts, it has been criticized that the needs of older adults are often neglected. This is based on the widespread assumption that bereavement may be less problematic in later life and that older adults would more readily accept loss [15]. Others have suggested that older adults may be more vulnerable to PGD than younger age groups due to smaller social networks and comorbid chronic illnesses [5]. In fact, several studies show older age to be associated with a higher risk for PGD [4,16], in addition to female sex, lower income, and loss of a spouse or a child [4]. However, many older adults with mental health problems do not receive psychotherapeutic or psychosocial treatment [17]. Various factors, such as fear of stigmatization or immobility, may be possible reasons for this. At the same time, older adults have become more open to using psychosocial support services in recent years [18]. In addition, the demand for accessible and efficient health care provision is becoming increasingly critical in light of the ongoing sociodemographic change. Projections indicate that by 2030, individuals aged 60 years and older will constitute approximately 35% of the German population [19]. As the proportion of older people increases, the demand for health care services, including medical care, psychosocial support, and therapeutic interventions, is expected to rise correspondingly [20].

The last 15 years have seen a surge of health care interventions provided through web-based programs or smartphone apps, specifically in the area of mental health. Such eHealth interventions have the advantage of a flexible and anonymous use, the possibility to reduce fear of stigmatization, provide a first contact with psychosocial care, and a higher outreach than traditional health care services. eHealth interventions based on cognitive behavioral therapies (CBTs) are among the most researched interventions in this field to date. There is broad evidence for the effectiveness of CBTs delivered via web-based or smartphone apps for various disorders, such as depression, anxiety, posttraumatic stress disorder (PTSD), or substance abuse [21-24]. With regard to prolonged grief, there are some studies showing promising results for interventions aiming to normalize grief, as well as for interventions focusing on the loss of a child during pregnancy and the loss of first-degree relatives [25-27]. Although the likelihood of experiencing loss and the risk of developing PGD increases in later life, there are only a few eHealth interventions focusing specifically on coping with grief in older adults [28,29]. Most eHealth interventions targeting grief were delivered via the internet, incorporated some form of therapist support or guidance, and consisted of 5 to 18 sessions or writing assignments [25-29]. Overall, there is considerable variation in the content structure of the interventions, ranging from writing assignments and psychoeducation to interactive exercises and exposure-based components. Notably, 1 study investigated an unguided, online intervention comprising 5 structured content modules and found significant improvements regarding self-efficacy, attitudes, and state anxiety [25]. Considering previous research in the field, it becomes evident that developing an eHealth intervention for prolonged grief requires a careful balance between delivering comprehensive informational and therapeutic content while also taking into account users’ attentional capacities and motivational constraints. This study reports the results of a randomized controlled trial (RCT) from the AgE-health project. AgE-health is a comprehensive research project aimed at developing and testing the web-based intervention, trauer@ktiv, focusing on loss and bereavement in later life. The eHealth intervention is based on established CBTs and has been developed by mental health care professionals (eg, psychologists, psychotherapists, and psychiatrists) with the involvement of older adults with loss experiences. The acceptance of the intervention was assessed before the trial using a qualitative approach [30]. We found high acceptance and good operability of use from both the perspective of the target group and of mental health care experts working in the field of geriatrics [30]. Trauer@ktiv is an unguided intervention. As such, users work through the program without therapeutic support. Unguided eHealth interventions illustrate the classic self-management approach and have been shown to be effective in treating depression and achieving similar effect sizes comparable to those of guided interventions in long-term follow-ups [31]. In the context of eHealth, self-management refers to interventions that enable individuals to take an active role in managing their own health issues [32]. Furthermore, older adults may be particularly suited for such interventions. A study testing an unguided, web-based self-management app for depressive symptoms reported higher adherence rates among older adults compared to younger participants [33]. Unguided eHealth interventions offer low-threshold interventions for mental health struggles, can account for logistical obstacles and mobility problems, and thus pave the way for seeking professional help.

### Objectives

This study aimed to assess the efficacy and acceptability of the web-based intervention, trauer@ktiv, to reduce prolonged grief symptoms in a sample of older bereaved individuals. The trauer@ktiv intervention focused on loss in later life and associated symptoms of grief reactions.

## Methods

### Study Context Information

The AgE-health study is a 2-arm, parallel-group RCT comparing an intervention group (IG) with an active control group (CG). The study was registered with the German Clinical Trials Register (DRKS00020595) and has been described in detail in the associated study protocol [34]. Trial results were reported in accordance with the CONSORT-EHEALTH (Consolidated Standards of Reporting Trials of Electronic and Mobile Health Applications and Online Telehealth) statement [35] (Multimedia Appendix 1).

### Ethical Considerations

The ethics committee of the Faculty of Medicine at Leipzig University, Germany, approved the study (ID 052/20-ek). Participants received comprehensive information about the AgE-health study, study aims, study procedure, and data security according to legal standards. Written informed consent was obtained from all participants before study participation. The AgE-health study adhered to the Declaration of Helsinki and the International Council for Harmonization Good Clinical practice Guidelines [36]. Furthermore, the study implemented an external data monitoring committee (DMC) to ensure that the study was performed in accordance with the Guidelines of Good Clinical Practice. The task of the DMC was to monitor protocol adherence, study safety, and study progress. Participants received an incentive of €30 (approximately US $34.9) for their study participation. To safeguard data privacy during the use of the web-based intervention and the transmission of metadata, anonymous log files were secured using Secure Sockets Layer technology. Passwords were stored in hashed form using the BCrypt algorithm, and all textual data entries were encrypted with AES-256 encryption via OpenSSL (the OpenSSL Project). Participants were free to discontinue study participation at any time. Access to the data was restricted to the research team.

### Sample Recruitment

The study sample was recruited through multiple pathways, including the publication of articles and study calls in local newspapers, distribution of flyers and posters in cemeteries and medical practices, and contacting support groups and older adult clubs. Furthermore, study calls were posted online via Facebook, advertisements (formerly “eBay Kleinanzeigen”), and mental health forums. Additional recruitment strategies included reaching out to former participants from previous studies at the research facility, conducting a local radio interview, and approaching church communities and hospices. Recruitment took place from September 2020 to May 2022. Interested individuals contacted the AgE-health study team. Consent papers, a screening assessment, and detailed information about the AgE-health study were delivered by postal mail to interested individuals. After the return of the consent form and completion of the screening assessment, individuals were informed of their inclusion or exclusion in the study. Individuals excluded from study participation received additional information on grief, mental health, and professional help.

### Inclusion and Exclusion Criteria

Study inclusion and exclusion criteria are provided in Textbox 1 [37-39].

**Inclusion and exclusion criteria.**

**Inclusion criteria**

- Experience of the death of a close person
- A period of 6 months or longer since the loss
- A score of ≥2 on the Brief Grief Questionnaire [37]
- Aged ≥60 years
- Internet access
- Sufficient proficiency in the German language

**Exclusion criteria**

- Suicidal tendencies (a score of ≥2 on the suicidality item of the Beck Depression Inventory [38,39])
- No contact person in case of a crisis (general practitioner or other medical specialist)
- Current psychotherapy
- Unstable psychopharmacological treatment (changes of medication within the last 6 weeks)
- Having had a severe mental disorder not related to bereavement that needed medical treatment within the last year

### Randomization and Blinding

Eligible participants were sequentially randomized to the IG or CG. Randomization was performed using a block randomization algorithm with an allocation ratio of 1:1, stratified by sex and grief severity to ensure balanced sample sizes in both allocation groups and to control for relevant covariates. Randomization block lists were generated by an independent statistician external to the study team. The sequential group allocation, according to the generated randomization lists, was carried out by a staff member external to the study team. Furthermore, block lists were coded to conceal the strata identification from the person responsible for the group allocation. Finally, the data analyst (AP) was blinded to the group allocation.

### Assessment Procedure

Eligible participants were assessed before receiving access to the intervention or bibliotherapy and before being notified of their group allocation (baseline assessment), as well as 4 months after the baseline assessment (follow-up), using comprehensive questionnaires sent by postal mail. Up to 3 postal reminders were sent in cases of missing returns.

### Interventions

#### The trauer@ktiv Intervention

Participants of the IG received free access to trauer@ktiv, a web-based intervention. Trauer@ktiv is an unguided and self-management promoting program designed to alleviate symptoms of prolonged grief and prevent late-life depression in grieving older adults. Trauer@ktiv was developed by mental health care professionals and is based on established CBTs. The intervention focuses specifically on adults aged 60 years and older, including respective example characters leading through the program. The intervention consists of 8 modules and has been described in detail in the study protocol [34]. Participants of the IG were advised to use the intervention regularly and work through 1 module per week. However, participants were free to use the intervention according to their own needs. In order to improve usability and ensure consistency in the implementation of the intervention, an instructional manual was provided within the trauer@ktiv intervention and was accessible to participants upon log-in.

#### Bibliotherapy

Participants of the CG received psychoeducational material in the form of an information brochure. The bibliotherapy consisted of 8 chapters providing general information on symptoms of grief, prolonged grief, as well as coping strategies and access to professional help. The study protocol provides an overview of the contents of the information brochure [34].

### Outcomes and Measures

The measurement instruments and outcome variables of the trial have been described in detail in the study protocol [34].

#### Screening Assessment

The screening assessment included questions on sociodemographic characteristics (age and sex) and questions to assess the eligibility criteria outlined in Textbox 1. The screening questionnaire further asked for the name and address of a medical contact person in case of a suicidal crisis. Individuals eligible for study participation underwent a comprehensive assessment regarding loss and bereavement, grief, and psychosocial health at baseline and follow-up.

#### Primary Outcome

The primary outcome was prolonged grief at follow-up, measured by the Prolonged Grief-13 scale (PG-13 [40,41]). The PG-13 assesses self-reported symptoms of prolonged grief and has been shown to have good psychometric properties (Cronbach α=0.94, test-retest reliability was 0.80 [40]). The instrument consists of 11 items measuring typical grief symptoms (eg, yearning and trouble accepting the loss) on a 5-point Likert scale. Two additional items assess functional impairments and the duration of the grief symptoms. A total score was calculated (range 11-55), with higher scores indicating more severe grief symptoms [42].

#### Secondary Outcomes

Secondary outcomes included the assessment of grief symptoms on 5 dimensions using the Würzburger Trauerinventar (WÜTI [43]). The WÜTI is a 24-item scale, rated on a 4-point Likert scale, covering the following 5 subscales: impairment, growth, guilt, empathy, and closeness to the deceased. Raw scores for each subscale are converted into percentile ranks based on gender-specific normative values [43]. Symptoms of depression were assessed with the 21-item Beck Depression Inventory, Second Edition (BDI-II [38,39]). In this study, a sum score was calculated (range 0-63). Higher scores indicated more severe symptoms of depression [38,39]. Participants’ social integration was measured with the Social Adaptation Self-Evaluation Scale, a 20-item scale assessing social activity [44,45]. Each item was rated on a 0 to 3 scale, resulting in a total score ranging from 0 to 60. Higher scores illustrated a higher degree of social integration [44,45]. Furthermore, social isolation was assessed using the 6-item Lubben Social Network Scale [46]. The total score ranged from 0 to 30, with a cutoff value less than 12 indicating an increased risk of social isolation [46]. The 12-item Short-Form Health Survey (SF-12 [47]) was used to measure health-related quality of life [47]. The SF-12 is a brief version of the 36-item Short-Form Health Survey, comprising 12 items rated on a 5-point Likert scale. The SF-12 measures quality of life across 2 main dimensions: physical and mental health. In each dimension, higher scores reflect a higher health-related quality of life [47]. Loneliness was measured with the 6-item De Jong Gierveld Loneliness Scale [48], which assesses both emotional and social loneliness on a 5-point Likert scale. Higher scores correspond to a higher degree of loneliness [48]. Finally, self-efficacy was assessed with the hope and self-efficacy subscale from the questionnaire for the Assessment of Empowerment in Patients with Affective and Schizophrenic Disorders [49]. A sum score was calculated (range 0-24), with higher scores indicating a higher degree of self-efficacy [49].

#### Assessments of Uptake, Acceptability, and Satisfaction

The follow-up assessment included additional questions on the satisfaction and uptake of the intervention or bibliotherapy. The 10-item System Usability Scale [50] was used as a measure of the acceptability of the eHealth intervention [50]. A total score (range 0-100) was calculated, with scores more than 80 indicating good to excellent usability [50]. In addition, pseudonymized metadata on the use of the eHealth intervention (log-in data and the access date of content modules) were collected. Engagement with the web-based intervention, trauer@ktiv, was operationalized as the total number of modules completed by the user. For each of the 8 modules, timestamps were recorded corresponding to the user’s access of the first and the last page. A module was considered completed if timestamps were available both for the first and for the last page, indicating that the user had accessed the entire module. With regard to the bibliotherapy, participants were asked to indicate whether they had engaged with the information brochure (response options: yes or no) and, if applicable, to report the frequency of their engagement.

#### Other Measures and Covariates

Furthermore, basic sociodemographic information (age, sex, marital status, education, living status, height, and weight), as well as information on religious belief, type of loss and time of loss, the use of health care and antidepressant drugs, internet use and internet familiarity, and personality characteristics (Big-Five Inventory–short form [51]) were assessed. Education was classified into three categories: low, middle, and high. **Low educational attainment** refers to individuals without a formal qualification or those with a lower secondary school certificate (typically completed by 9th grade), with or without vocational or semiskilled training. **Middle educational attainment** refers to individuals with an intermediate secondary school certificate (completed by 10th grade) or a high school diploma, with or without additional vocational or semiskilled training. **High educational attainment** encompasses individuals who have completed a degree at a university of applied sciences, an engineering school, or a university.

### Data Management and Quality Control

Data were collected in a pseudonymous form and entered in a database using the statistical software SPSS (version 27.0, IBM Corp). A double-entry check was performed to ensure the completeness and accuracy of data entry. In addition, a commissioned external statistician conducted data auditing. Auditing consisted of reviewing the data collection to check its consistency with the electronic data file and included the inspection of 5% of the questionnaires at baseline and follow-up. The questionnaires were randomly drawn and inspected.

### Sample Size

On the basis of previous comparable trials using the PG-13 as primary outcome [26,28,52], a moderate effect size of Cohen *d*=0.5 was considered to power the trial. Given a significance level of α=.05 and a statistical power of 1–β=.8, the optimal sample size to detect a moderate between-group effect at 4-month follow-up was calculated to be 128 (n=64, 50% per group). Considering a dropout rate of 25% based on previous trials [53,54], an initial sample size of 170 participants at baseline was aimed for.

### Statistical Analysis

Data quality checks showed a generally low frequency of missing information (ie, 8/170, 4.7% of the cases or less) for most variables at baseline; however, the frequencies were higher for BDI-II (12/170, 7.1%) and the WÜTI subscale growth (10/170, 5.9%), and highest for the SF-12 (15/170, 8.8%). Cases with missing data were excluded from the analyses.

Intention-to-treat (ITT) analyses were performed to evaluate the efficacy of the intervention with regard to primary and secondary outcomes. Descriptive data were presented as case numbers with percentages or means with SDs or 95% CIs. Treatment effects at 4-month follow-up were analyzed using mixed-effects linear regression models for all outcomes except the De Jong Gierveld Loneliness Scale, for which mixed-effects tobit regression models were used. All models included group, time, and the group-by-time interaction as fixed effects; a random intercept to account for patient-level heterogeneity; and adjustments for sociodemographic characteristics (age, sex, and education) and relevant covariates (professional treatment for grief symptoms and psychotropic medication). Treatment effects in outcomes were shown as average marginal effects with 95% CIs, which indicate the sample average of the estimated group difference in an outcome at follow-up. The Huber White sandwich estimator was used to estimate SEs in all models.

Statistical analyses were conducted using SPSS Statistics (version 27) and Stata (version 16.1 Special Edition; StataCorp LLC) software package. The level of statistical significance was set at *P*<.05 for all analyses.

## Results

### Participants

**Figure 1:**
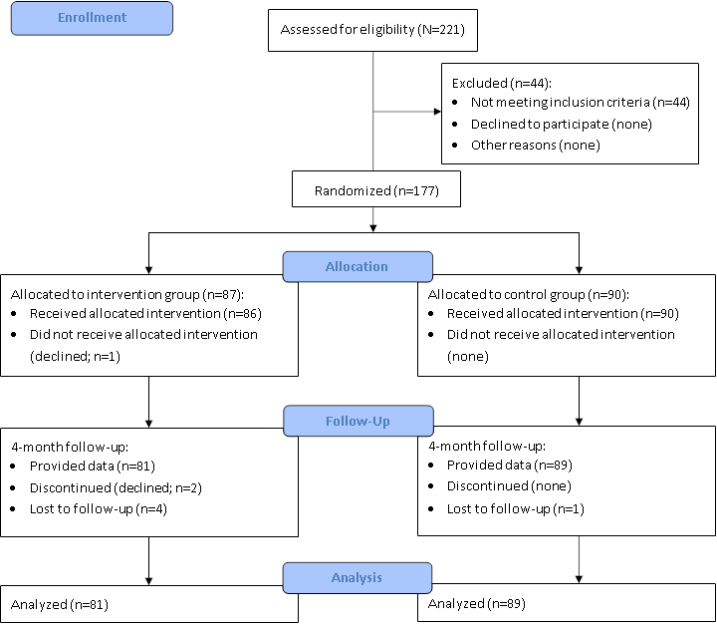
Flowchart of sample selection and randomization in the AgE-health trial.

An overview of the participant flow is shown in Figure 1. In total, 275 interested individuals contacted the study team. Of those, 80.4% (221/275) were screened for eligibility. In most cases, individuals became aware of the study via newspaper articles and study calls published in newspapers (117/221, 52.9%). Furthermore, awareness of the AgE-health study through postings on cemeteries (28/221, 12.7%), family or friends (23/221, 10.4%), and the internet (22/221, 10%) contributed with similar proportions. Further avenues through which participants became aware of the study comprised medical practices (8/221, 3.6%), support groups (6/221, 2.7%), and hospices (5/221, 2.3%), in addition to a range of other access ways (eg, churches and senior citizen clubs; 12/221, 5.4%). Of those screened, 177 were eligible for study participation and randomized to either IG (n=87, 49.2%) or CG (n=90, 50.8%). Most frequently, individuals were excluded from study participation due to a current psychotherapy treatment (25/221, 11.3%). Other reasons for exclusion were age (<60 years; 2/221, 0.9%), lack of internet access (8/221, 3.6%), a score of less than 2 on the Brief Grief Questionnaire (2/221, 0.9%), suicidality (4/221, 1.8%), lack of access to a medical expert (2/221, 0.9%), having had a severe mental disorder in the last year (7/221, 3.2%), and recent changes in psychotropic drug use (2/221, 0.9%). Of those randomized, 4.1% (7/177) of the participants were lost to follow-up. Finally, 170 individuals were included in the analysis. Included and excluded individuals did not differ with regard to age (included individuals: mean 67.3, SD 6.4 y vs excluded individuals: mean 67.1, SD 7.4 y; 2-tailed t_219_=–0.179; *P*=.86) and sex (included individuals: 129/170, 75.9% female vs excluded individuals: 42/51, 82% female; *χ*^2^_1_=0.9; *P*=.33). However, excluded individuals had a higher symptom severity on the Brief Grief Questionnaire (included individuals: mean 6.7 vs excluded individuals: mean 7.3; 1-tailed t_219_=1.86; *P*=.03) and were prescribed psychotropic drugs more frequently (included individuals: 19/170, 11.2% vs excluded individuals: 14/51, 28%; *χ*^2^_1_=8.2; *P*=.007).

Sociodemographic characteristics for the total sample, IG, and CG are provided in Table 1. The recruited sample was aged on average 67.3 (SD 6.4) years, predominantly female (129/170, 75.9%), widowed (87/170, 51.2%), and living alone (117/170, 68.8%). Half of the AgE-health sample had a high (87/170, 51.2%) or a middle educational status (80/170, 47.1%), and only 1.8% (3/170) had a low educational status. Less than one-third (53/170, 31.2%) of the sample was working, and less than one-quarter (39/170, 22.9%) belonged to a church. Participants of the IG and CG did not differ in their sociodemographic characteristics. With regard to the primary outcome, the mean score on the PG-13 was 31.15 and 31.38 in the IG and CG, respectively.

The primary and secondary outcomes at baseline for both treatment groups are provided in Table 2.

**Table 1:** Sociodemographic characteristics of the AgE-health sample (N=170).

Characteristics		Total sample	Intervention group (n=81)	Control group (n=89)
Age (y), mean (SD; range)		67.3 (6.4; 60-95)	67.0 (5.1; 60-83)	67.6 (7.3; 60-95)
**Sex, n (%)**				
	Female	129 (75.9)	63 (77.8)	66 (74.2)
	Male	41 (24.1)	18 (22.2)	23 (25.8)
**Marital status, n (%)^a^**				
	Married or in partnership	38 (22.4)	18 (22.2)	20 (22.5)
	Divorced or separated	30 (17.6)	15 (18.5)	15 (16.9)
	Widowed	87 (51.2)	43 (53.1)	44 (49.4)
	Single	13 (7.6)	4 (4.9)	9 (10.1)
Living alone, n (%)^b^		117 (68.8)	53 (65.4)	64 (71.9)
**Educational status, n (%)^c^**				
	High	87 (51.2)	43 (53.1)	44 (49.4)
	Middle or low	83 (48.8)	38 (46.9)	45 (50.6)
Working, n (%)^d^		53 (31.2)	21 (25.9)	32 (36)
Belonging to a church, n (%)		39 (22.9)	16 (19.8)	23 (25.8)
BMI, mean (SD)^e^		27.1 (5.5)	27.1 (5.4)	27.1 (5.6)

^a^Missing data of 2 individuals.

^b^Missing data of 1 individual.

^c^Middle and low educational status are reported in 1 category as only 1.8% (3/170) of the total sample had a low educational status. Low educational status: individuals without a formal qualification or those with a lower secondary school certificate (typically completed by 9th grade), with or without vocational or semiskilled training; middle educational status: individuals with an intermediate secondary school certificate (completed by 10th grade) or a high school diploma, with or without additional vocational or semiskilled training; high educational status: individuals who have completed a degree at a university of applied sciences, an engineering school, or a university.

^d^Included individuals are those with a pension or unemployment status who have a minor job simultaneously.

^e^Missing data of 5 individuals.

**Table 2:** Primary and secondary outcomes at baseline for the intervention group (IG) and control group (CG; N=170).

Outcome				CG (n=89)					IG (n=81)			
				Participants, n (%)		Values, mean (95% CI)		Participants, n (%)				Values, mean (95% CI)
**Primary outcome**												
	PG-13^a^ sum score											
		Baseline			89 (100)		31.38 (29.39-33.36)			78 (96)	31.15 (29.06-33.23)	
		4 months			85 (96)		29.28 (27.16-31.39)			79 (98)	29.16 (26.88-31.44)	
**Secondary outcomes**												
	**Grief symptoms (WÜTI^b^** **)**											
		**Impairment**										
			Baseline		83 (93)		47.34 (41.35-53.34)			79 (98)	47.91 (41.43-54.38)	
			4 months		87 (98)		40.21 (34.12-46.29)			76 (94)	38.47 (32.20-44.75)	
		**Growth**										
			Baseline		84 (94)		46.88 (41.21-52.54)			79 (98)	47.51 (41.80-53.22)	
			4 months		85 (96)		50.25 (44.61-55.88)			75 (93)	53.09 (47.80-58.38)	
		**Guilt**										
			Baseline		85 (96)		61.90 (56.01-67.79)			79 (98)	63.01 (56.96-69.05)	
			4 months		85 (96)		54.31 (48.32-60.30)			78 (96)	53.53 (47.27-59.78)	
		**Empathy**										
			Baseline		86 (97)		55.53 (49.35-61.71)			78 (96)	47.88 (41.22-54.54)	
			4 months		86 (97)		55.53 (49.20-61.87)			77 (95)	50.22 (43.98-56.46)	
		**Closeness**										
			Baseline		87 (98)		62.43 (56.71-68.15)			79 (98)	64.54 (58.33-70.75)	
			4 months		85 (96)		58.54 (52.42-64.66)			77 (95)	57.30 (50.72-63.87)	
		**Depression (BDI^c^** **)**										
			Baseline		81 (91)		15.67 (13.65-17.70)			77 (95)	16.46 (14.38-18.54)	
			4 months		82 (92)		13.45 (11.55-15.35)			77 (95)	13.64 (11.59-15.68)	
	**Loneliness (DJGLS^d^** **)**											
		**Emotional loneliness**										
			Baseline		87 (98)		2.52 (2.36-2.68)			80 (99)	2.38 (2.19-2.57)	
			4 months		84 (94)		2.46 (2.29-2.63)			80 (99)	2.29 (2.11-2.47)	
		**Social loneliness**										
			Baseline		87 (98)		2.13 (1.95-2.30)			81 (100)	2.07 (1.91-2.24)	
			4 months		83 (93)		2.09 (1.91-2.26)			80 (99)	2.09 (1.90-2.27)	
	**Social activity (SASS^e^** **)**											
		Baseline			86 (97)		39.69 (38.09-41.29)			76 (94)	40.89 (39.33-42.45)	
		4 months			81 (91)		41.01 (39.22-42.80)			77 (95)	41.44 (39.55-43.34)	
	**Social isolation (LSNS^f^)**											
		Baseline			88 (99)		15.43 (14.21-16.64)			79 (98)	15.58 (14.40-16.75)	
		4 months			88 (99)		15.27 (14.01-16.54)			80 (99)	15.79 (14.56-17.01)	
	**Self-efficacy (EPAS^g^)**											
		Baseline			86 (97)		13.76 (12.74-14.79)			79 (98)	14.36 (13.41-15.31)	
		4 months			84 (94)		14.32 (13.30-15.35)			80 (99)	15.21 (14.20-16.22)	
	**Quality of life (SF-12^h^** **)**											
		**Physical health**										
			Baseline		80 (90)		42.99 (40.53-45.45)			75 (93)	44.37 (42.02-46.72)	
			4 months		78 (88)		43.23 (40.67-45.79)			73 (90)	44.60 (42.27-46.94)	
		**Mental health**										
			Baseline		80 (90)		41.02 (38.71-43.34)			75 (93)	40.15 (37.65-42.66)	
			4 months		78 (88)		42.04 (39.52-44.56)			73 (90)	44.05 (41.29-46.80)	

^a^PG-13: Prolonged Grief-13 scale.

^b^WÜTI: Würzburger Trauerinventar. Reported scores were percentile ranks: WÜTI acute emotional and cognitive impairments subscale, WÜTI general personality development and growth subscale, WÜTI feelings of guilt or self-reproaches subscale, WÜTI increase in sensibility or empathy for others subscale, and WÜTI closeness to the deceased subscale.

^c^BDI: Beck Depression Inventory.

^d^DJGLS: De Jong Gierveld Loneliness Scale.

^e^SASS: Social Adaptation Self-Evaluation Scale.

^f^LSNS: Lubben Social Network Scale.

^g^EPAS: Empowerment in Patients with Affective and Schizophrenic Disorders, hope and self-efficacy subscale.

^h^SF-12: Short-Form Health Survey.

### Attrition

In total, 3.9% (7/177) of the participants randomized did not complete assessments at 4-month follow-up. Of those, 6 participants belonged to the IG and 1 participant belonged to the CG. Two participants refused further participation in the trial. In 5 cases, assessment questionnaires were not returned despite repeated reminders. Sensitivity analyses showed no significant differences between participants lost to follow-up (7/177, 3.9%) and completers (170/177, 96%) regarding age (mean 67.1 vs mean 67.3; *z* score=–0.173; *P*=.87), sex (5/7, 71% vs 129/170, 75.9% female, participants; *χ*^2^_1_=0.1; *P*=.79), or symptom severity on the primary outcome (PG-13: mean 33.3 vs mean 31.3; t_172_=0.536; *P*=.59) at baseline.

### ITT Analyses

Results of the ITT analyses are provided in Table 3. No significant differences were found between the IG and CG in the primary outcome at 4-month follow-up (PG-13: average marginal effect 0.56, 95% CI –2.30 to 3.44). Similarly, there were no significant differences for secondary outcomes between the IG and CG at follow-up.

**Table 3:** Results of the AgE-health trial based on the intention-to-treat analysis: between-group differences in primary and secondary outcomes at 4-month follow-up (N=170).

Outcome				Participants, n (%)		AME^a^ (95% CI)		*P* value
**Primary outcome**								
	PG-13^b^ sum score			169 (99.4)		0.56 (−2.30 to 3.44)		.70
**Secondary outcomes**								
	**Grief symptoms (WÜTI^c^** **)**							
		Impairment	169 (99.4)		0.85 (−7.33 to 9.04)		.84	
		Growth	168 (98.8)		1.67 (−5.82 to 9.17)		.66	
		Guilt	168 (98.8)		−0.84 (−9.12 to 7.43)		.84	
		Empathy	168 (98.8)		−5.32 (−13.99 to 3.34)		.23	
		Closeness	169 (99.4)		−0.15 (−8.62 to 8.31)		.97	
	Depression (BDI^d^)			169 (99.4)		0.29 (−2.16 to 2.75)		.81
	**Loneliness (DJGLS**^e^)							
		Emotional loneliness	170 (100)		−0.20 (−0.47 to 0.07)		.15	
		Social loneliness	170 (100)		0.02 (−0.27 to 0.32)		.88	
	Social activity (SASS^f^)			169 (99.4)		0.32 (−2.16 to 2.81)		.80
	Social isolation (LSNS^g^)			169 (99.4)		0.27 (−1.45 to 2.00)		.76
	Self-efficacy (EPAS^h^)			170 (100)		0.73 (−0.64 to 2.10)		.30
	**Quality of life (SF-12^i^)**							
		Physical health	166 (97.6)		1.13 (−2.13 to 4.39)		.50	
		Mental health	166 (97.6)		1.34 (−2.04 to 4.74)		.44	

^a^AME: average marginal effect; all models were adjusted for age, sex, education, professional treatment for grief symptoms, and psychotropic medication.

^b^PG-13: Prolonged Grief-13 scale.

^c^WÜTI: Würzburger Trauerinventar. Reported scores were percentile ranks, WÜTI acute emotional and cognitive impairments subscale, WÜTI general personality development and growth subscale, WÜTI feelings of guilt or self-reproaches subscale, WÜTI increase in sensibility or empathy for others subscale, and WÜTI closeness to the deceased. subscale

^d^BDI: Beck Depression Inventory.

^e^DJGLS: De Jong Gierveld Loneliness Scale.

^f^SASS: Social Adaptation Self-Evaluation Scale.

^g^LSNS: Lubben Social Network Scale.

^h^EPAS: Empowerment in Patients with Affective and Schizophrenic Disorders, hope and self-efficacy subscale.

^i^SF-12: Short-Form Health Survey.

### Per-Protocol Analyses and Response and Remission Rates

Per-protocol analyses involving participants in the IG who accessed the trauer@ktiv intervention (IG: 69/158, 43.7% and CG: 89/158, 56.3%) showed no significant between-group effects at follow-up for primary and secondary outcomes. In addition, remission frequency, defined as PG-13 <35 according to Pohlkamp et al [55], showed no significant difference between IG and CG at 4-month follow-up (remission in CG: 6/28, 21% and IG: 6/27, 22%). Similarly, response rates, defined as a decrease of 33% in symptom severity on the PG-13 from baseline to follow-up, showed no statistical difference between IG and CG (33% response in CG: 6/85, 7% and IG: 4/77, 5%). Of the 81 participants randomized to the IG, 51 (63%) improved in their grief intensity, 6 (7%) experienced no change, and 20 (25%) showed symptom intensification on the PG-13 from baseline to follow-up. With regard to the 89 participants in the CG, 55 (62%) improved in their grief intensity, while 3 (3%) experienced no change and 27 (30%) showed an increased grief intensity on the PG-13 from baseline to follow-up. Improvements on the PG-13 were, on average, 4.9 (range 1-18) points in the IG and 4.8 (range 1-18) points in the CG. Symptom intensification was, on average, 4.0 (range 1-11) points in the IG and 3.4 (range 1-9) points in the CG.

### Uptake, Adherence, and Acceptability of the Intervention

In total, 85% (69/81) of the participants of the IG stated that they had accessed trauer@ktiv during the intervention period. In addition to the self-reported data, the pseudonymized log-in data were evaluated. More than half (49/81, 61%) completed 5 or more modules, and further 22% (18/81) completed between 1 and 4 modules of trauer@ktiv. A total of 28% (23/81) of the participants completed all 8 modules of the intervention trauer@ktiv. Analysis of the System Usability Scale showed an average sum score of 81.0 (SD 15.8), indicating an overall good usability for the intervention. Information on satisfaction with the intervention was provided by 84% (68/81) of the respondents of the IG as part of the follow-up assessment. Of these 68 respondents, 63 (93%) were satisfied or very satisfied with trauer@ktiv, 4 (6%) were dissatisfied, and 1 (2%) were very dissatisfied. Those who did not test the intervention (12/81, 15%) most frequently stated that they could not yet bring themselves to do it (5/81, 6%); they experienced technical problems with their internet connection, their device, or the intervention (5/81, 6%); or they lacked motivation to test trauer@ktiv (4/81, 5%).

Regarding the bibliotherapy, 78 (88%) of the 89 participants of the CG stated that they had read the information brochure. Of these 78 participants, 60 (77%) were satisfied or very satisfied, 14 (18%) were dissatisfied, and 3 (4%) were very dissatisfied with the bibliotherapy. Those who did not read the information brochure (11/89, 12%) most frequently stated that they lacked motivation to read the bibliotherapy (7/89, 8%), they could not yet bring themselves to read it (4/89, 5%), or they had doubts as to whether the bibliotherapy would be helpful to them (3/89, 3%).

### Harms

Throughout the intervention period, 1 adverse event (AE) with a possible causal link to the web-based intervention, trauer@ktiv, was recorded when a participant of the IG reported an increase in distress after inappropriate intensive use of the intervention. The participant had completed all program modules of trauer@ktiv in quick succession, contrary to the study team’s recommendation, and subsequently experienced a temporary increase in emotional distress. The AE was countered with supportive talks conducted by a psychotherapist (FW) via telephone. The discussion focused on ways of relieving stress, using the personal and professional support system, and pausing the program. In total, 3 monitoring checkups were administered. Supportive talks led to a noticeable relief in stress symptoms. The participant remained in the trial and took part in the follow-up assessment. The AE was documented using an AE or severe AE protocol form and was continuously reviewed by 2 principal investigators (ML and SGR-H) and the DMC.

## Discussion

### Principal Findings

This study aimed to test the efficacy of the web-based intervention, trauer@ktiv, at reducing prolonged grief symptoms in a sample of older adults. Contrary to the expectation, ITT analyses showed no significant differences between the IG and CG for the primary outcome or any of the secondary outcomes at the 4-month follow-up assessment. Similarly, no significant group differences were found using per-protocol analyses. On average, both IG and IG demonstrated a reduction of 2 points in symptom severity on the primary outcome. Furthermore, no between-group differences were found in terms of remission or response rates. However, uptake of and adherence to the eHealth intervention were overall satisfactory, with more than half (49/81, 61%) of the IG completing 5 modules or more. Of those who accessed the intervention, more than 92% were satisfied or very satisfied with it. There was 1 AE linked to an inappropriate excessive use of the intervention trauer@ktiv.

This study examined the superiority of an unguided eHealth intervention over a bibliotherapy in adults aged 60 years and above who had experienced the loss of a close person. Its findings contrast with previous research reporting the efficacy of eHealth interventions targeting grief symptoms or prolonged grief [28,29,56-58]. Discrepancies in findings may be partly explained by methodological differences across studies, such as variations in sample characteristics, level of guidance, and CG design. Litz et al [29] developed and tested a therapist-assisted web-based intervention in recently bereaved adults. Participants were caregivers who had lost a loved one 3 months to 1 year before the study inclusion. The intervention focused on self-care, wellness, and behavioral activation and proved to be effective in reducing prolonged grief symptoms compared to a waitlist CG [29]. Similarly, Brodbeck et al [28] reported significant improvements with regard to grief, depression, distress, and loneliness for a guided eHealth intervention aimed at widowed older adults and individuals after separation or divorce. However, only 23% of the sample were older adults after spousal bereavement. Most of the sample consisted of adults who had experienced separation or divorce [28]. Generally, both studies included mostly younger older adults, with an average sample age of 51 [28] and 55 [29] years. In comparison, participants in this study had an average age of 67 years, ranging from 60 to 95 years. It is important to make this distinction. Younger older adults are often still working and have a larger social network and therefore more social resources compared to older adults aged more than 65 years, who are retired and may have to cope with multiple loss experiences, greater social isolation, and chronic health issues. Furthermore, both studies compared the intervention to a waitlist rather than an active CG. RCTs comparing an IG to a waitlist control have been criticized for producing exaggerated treatment effects and a possible nocebo effect [59,60]. Generally, methodological issues are important to consider when comparing RCT results on the effectiveness of digital interventions. Zuelke et al [58] provided a systematic review and meta-analysis on the effectiveness of web-based interventions targeting grief, including prolonged or complicated grief. The authors further assessed the overall quality of the included studies and the applied web-based interventions. While the meta-analysis showed moderate effect sizes for reducing grief and depression and a large effect size for reductions in PTSD symptoms, the analysis further indicated publication bias, specifically for interventions on grief and depression. In their discussion, the authors evaluated the overall quality of existing evidence on digital grief interventions as low for the reduction of grief symptoms and depression, and moderate for PTSD [58]. Few studies have reported mixed results with regard to the effectiveness of web-based self-help interventions [61,62]. Tur et al [61] tested a web-based CBT for prolonged grief in a small sample of 6 younger adults and found inconclusive results. Only half of the sample benefited in terms of experiencing a clinically significant reduction in grief symptoms [61].

There are several possible reasons for the nonsignificant results in this study. First, the implementation of the trial fell entirely within the period of the COVID-19 pandemic; however, the trauer@ktiv intervention had been developed and finalized just before the pandemic began. Thus, the intervention did not take into account the challenges posed by a pandemic. It is well known that the COVID-19 pandemic had a strong impact on grief and bereavement support. It has been suggested that grief may be more difficult to process during the pandemic because of associated factors, such as traumatic deaths, social isolation in the aftermath of a loss, higher tensions within families or households, a limited availability of health care, or an increased anxiety about contagion in social contacts [56,63]. While older adults have been shown to cope comparatively well with such difficulties during that time [64], the COVID-19 pandemic specifically disrupted certain grief rituals and the possibilities to say farewell to the deceased. In line with those challenges, studies have reported high needs for emotional support of bereaved individuals and expressed concern for an increased risk of developing PGD during the COVID-19 pandemic [65,66]. Therefore, it can be assumed that the COVID-19 pandemic itself may have negatively affected individuals’ ability to cope with their loss, even when provided with an intervention. Furthermore, the trauer@ktiv intervention has a strong focus on behavioral activation and the establishment of new social relationships or the resuming of previous ones. Because such activities were strictly limited during the COVID-19 pandemic to avoid further contagion in the population, the mismatch between the intervention content designed to promote behavioral activation and the real-life conditions of social isolation may have been the most important reason for its lack of effectiveness. A review on web-based grief interventions found the highest attrition rate in a behavioral activation group in 1 study, suggesting that behavioral change may generally be more difficult to achieve by oneself [57]. Similarly, recent reviews have reported that web-based grief interventions appear to be more effective in reducing grief-related PTSD symptoms than in reducing grief symptoms per se [57,58]. In this respect, it may also be possible that the focus on behavioral activation in our study is in itself less effective than a stronger focus on exposure would have been. While one of the modules in trauer@ktiv specifically addressed the relevance of exposure to difficult emotions and memories, this may have been too low in intensity to achieve a meaningful effect. However, Litz et al [29] demonstrated the effectiveness of an eHealth intervention that focused mostly on self-care and behavioral activation and did not include any exposure sessions. Therefore, future studies should test for the specific contents of eHealth interventions that are effective in reducing grief severity and PTSD symptoms.

Another possible reason for the nonsignificance of the intervention tested in this study may be related to the level of guidance. We tested the efficacy of an unguided self-management intervention. Unguided eHealth interventions have been shown to be effective; however, they usually appear to have smaller effect sizes compared to guided interventions, specifically in the short term [31]. With respect to grief interventions, most eHealth applications tested in RCTs provided some form of minimal guidance or therapy support [28,29,57,58,67]. A higher degree of guidance and level of personal feedback provided to participants of such eHealth interventions for grief have been associated with lower attrition rates [57]. It has further been suggested that bereaved individuals experiencing prolonged grief may need more personal support to continue with the intervention [57], which is in line with previous studies reporting a high need for emotional and social support of the bereaved in general [63,65].

With regard to this study, the uptake of the trauer@ktiv eHealth intervention was high. Approximately 85% (69/81) of those included in the analysis and assigned to the IG logged into the intervention at least once. Previous studies have reported lower average uptake rates of 70% to 79% for digital mental health interventions [33,68]. Regarding adherence to trauer@ktiv, 60% (49/81) of the participants completed 5 or more modules, and 28% (23/81) completed all 8 modules of the intervention. Similar completion rates have been reported by a previous study testing the effectiveness of a web-based, unguided CBT for depression [33]. The authors report a completion rate of 27% for the oldest age group of adults aged ≥60 years. Interestingly, they found improved adherence with increasing age, as only 10% of the youngest (18-39 years) and 22% of the middle-aged (40-59 years) participants completed the intervention [33]. In another study, which investigated the effectiveness of a web-based, unguided intervention with a specific focus on loss during the COVID-19 pandemic, only 5.1% of those assigned to the IG completed the intervention [56]. Completion rates have been shown to vary substantially across studies [69]. Generally, adherence rates appear to be higher for guided compared to unguided eHealth interventions [28,70]. Still, the uptake and adherence to the eHealth intervention in this study were satisfactory for an unguided intervention, with more than half of the participants assigned to the IG completing more than half of the intervention. However, some form of low-level guidance, such as weekly feedback or reminders for those who do not progress, may be needed to further improve adherence and outcome effects, specifically in a sample of bereaved individuals.

Although there was no superiority of the trauer@ktiv eHealth intervention compared to an active CG, the results also show that most (57/81, 70%) participants did not deteriorate in their grief through using the eHealth intervention. Both treatment groups showed small, albeit not significant, improvements in grief symptom severity over the course of 4 months. As usability and satisfaction with the application were high among older bereaved individuals, trauer@ktiv may have potential within a stepped and collaborative treatment approach offering easy access to grief support independent of time or mobility constraints of the bereaved.

### Strengths and Limitations

The AgE-health study is characterized by various strengths, such as a robust RCT design, the use of validated instruments, low attrition rates, and adequate power. The robust RCT design, which included the stratified block randomization performed by an external statistician, allowed for balanced samples in the IG and CG at baseline. Both groups did not differ with regard to sociodemographic characteristics, the primary outcome, or relevant covariates at baseline. Furthermore, a rigorous data quality control was achieved through double data entry checks and an external data auditing of 5% of the questionnaires at baseline and follow-up, each. Another strength of the study was the implementation of an external DMC. The DMC monitored study safety, study progress, and the conduct of the study in accordance with the Guidelines of Good Clinical Practice. Generally, the tested intervention, trauer@ktiv, was safe and easy to use for the intended target group of older adults. On average, participants of the IG did not deteriorate in their grief severity through the use of the intervention. There was 1 mild AE linked to an inappropriate, excessive use of the eHealth intervention within a short time frame, contrary to the recommendation of the study team. Albeit being an unguided eHealth intervention, there was a satisfactory uptake and adherence to it by older adults. Furthermore, those who tested trauer@ktiv were mostly satisfied or very satisfied with the intervention. Another strength of this study was the comparison of the intervention against an active CG, thus reducing the risk of exaggerated treatment effects or bias of results through a possible nocebo effect.

There are also a few limitations to consider. First, the sample consisted predominantly of female participants who were well educated, thus limiting the generalizability of the results. This is a common problem with intervention studies in the field of mental health research and specifically with regard to grief interventions. There are several reasons for the overrepresentation of women in such studies. For one, in the population of older adults, women are generally more often widowed than men [71]. It has further been suggested that women may be more frequently involved in the care of sick or terminally ill family members, making them more susceptible to traumatic experiences with regard to the death of loved ones [67]. Finally, studies have shown women to be more open with regard to psychosocial support than men [72]. Furthermore, the absence of explicit considerations for cultural diversity during participant recruitment may have led to sample selection bias, thereby limiting the extent to which the trial’s findings can be generalized to the wider population of older adults in Germany. In addition, due to the nature of the trial, participants were not blinded to their group allocation, which may have introduced a potential expectation bias and potentially compromised the internal validity of the study findings. However, no significant differences were observed in either the primary or secondary outcomes between the IG and CG, and participants in both groups reported high levels of satisfaction with the trauer@ktiv intervention or the bibliotherapy. Thus, the likelihood of an expectation bias influencing the results appears to be low. With regard to the level of education, the nature of the study itself, testing an eHealth intervention, may have introduced a sample selection bias, as access to the internet was a prerequisite to participate in the trial. Another limitation to consider is the reliance on self-report assessments in this study rather than clinical diagnoses of PGD. However, in this study, prolonged grief was assessed using the PG-13, which is a validated instrument to assess PGD and has been shown to have good psychometric properties [40]. Similarly, self-report measures were used instead of clinical diagnoses to evaluate additional dimensions of psychosocial health (eg, BDI-II). While self-report instruments may be subject to bias related to recall or self-assessment of symptoms, only validated tools with robust psychometric properties were included in the study. As an additional limitation, no imputation methods were applied to address missing data. However, the frequency of missing data was relatively low, and a sensitivity analysis indicated that missingness was not associated with gender or educational level. Age emerged as the only variable associated with increased missingness, as older participants were more likely to provide incomplete data. Furthermore, the pattern of higher rates of missing data among older participants is consistent with previous research [73]. Furthermore, the COVID-19 pandemic and its associated restrictions on daily activities and social connectedness may have acted as a possible confounder on the treatment results, as the RCT was conducted entirely within the time frame of the pandemic.

### Conclusions

The tested eHealth intervention, trauer@ktiv showed no superiority in reducing prolonged grief symptoms in comparison to an active CG in a sample of bereaved older adults. Both treatment groups showed small improvements of similar size on the primary outcome 4 months after access to the eHealth intervention or the information brochure. The trauer@ktiv eHealth intervention was developed before the COVID-19 pandemic and relied partly on behavioral activation. However, measures to prevent contagion during the COVID-19 pandemic may have had a detrimental impact on the effectiveness of the eHealth intervention as the conduction of the trial fell completely within the time frame of the pandemic. On a positive note, the trauer@ktiv intervention showed high satisfaction ratings and can generally be considered safe and easy to use for older adults experiencing prolonged grief symptoms. Thus, trauer@ktiv may have potential in a stepped-care approach as a low-threshold intervention, defined as a readily accessible web-based intervention, which can be used without professional support or fixed time and place requirements. In light of the limited evidence on eHealth interventions targeting prolonged grief in older adults, the results of this study should be considered preliminary. Further research is essential to expand upon these results. Future studies should investigate which contents of grief interventions, such as behavioral activation or exposure, are most effective for prolonged grief in older adults when provided via web-based interventions. Furthermore, there is still a need for methodologically robust RCTs testing eHealth interventions against active CGs rather than waitlist conditions.

## Appendix

Multimedia Appendix 1 CONSORT-EHEALTH checklist (V 1.6.1).

## Abbreviations

AE: adverse event
BDI-II: Beck Depression Inventory, Second Edition
CBT: cognitive behavioral therapy
CG: control group
CONSORT-EHEALTH: Consolidated Standards of Reporting Trials of Electronic and Mobile Health Applications and Online Telehealth
DMC: data monitoring committee
DSM-5: Diagnostic and Statistical Manual of Mental Disorders (Fifth Edition)
ICD-11: International Statistical Classification of Diseases and Related Health Problems, 11th Revision
IG: intervention group
ITT: intention-to-treat
PG-13: Prolonged Grief-13 scale
PGD: prolonged grief disorder
PTSD: posttraumatic stress disorder
RCT: randomized controlled trial
SF-12: Short-Form Health Survey
WÜTI: Würzburger Trauerinventar

## Data Availability

The datasets generated or analyzed during this study are not publicly available due to privacy and ethical restrictions; however, they are available from the corresponding author on reasonable request.

## References

[1] American Psychiatric Association (2022). Diagnostic and Statistical Manual of Mental Disorders, Fifth Edition.

[2] International Classification of Diseases 11th revision: the global standard for diagnostic health information. World Health Organization.

[3] Comtesse H, Smid GE, Rummel AM, Spreeuwenberg P, Lundorff M, Dückers ML (2024). Cross-national analysis of the prevalence of prolonged grief disorder. J Affect Disord.

[4] Kersting A, Brähler E, Glaesmer H, Wagner B (2011). Prevalence of complicated grief in a representative population-based sample. J Affect Disord.

[5] Thiemann P, Street AN, Heath SE, Quince T, Kuhn I, Barclay S (2023). Prolonged grief disorder prevalence in adults 65 years and over: a systematic review. BMJ Support Palliat Care.

[6] Boelen PA (2015). Peritraumatic distress and dissociation in prolonged grief and posttraumatic stress following violent and unexpected deaths. J Trauma Dissociation.

[7] Groot MH, de Keijser J, Neeleman J (2006). Grief shortly after suicide and natural death: a comparative study among spouses and first-degree relatives. Suicide Life Threat Behav.

[8] Kochen EM, Jenken F, Boelen PA, Deben LM, Fahner JC, van den Hoogen A, Teunissen SC, Geleijns K, Kars MC (2020). When a child dies: a systematic review of well-defined parent-focused bereavement interventions and their alignment with grief- and loss theories. BMC Palliat Care.

[9] Bahrevar V, Khankeh H, Morowatisharifabad MA, Foroughan M, Rashedi V (2025). The grief of older parents over the loss of a child: a scoping review. Omega (Westport).

[10] Kaunonen M, Tarkka MT, Paunonen M, Laippala P (1999). Grief and social support after the death of a spouse. J Adv Nurs.

[11] Cacciatore J, Thieleman K, Fretts R, Jackson LB (2021). What is good grief support? Exploring the actors and actions in social support after traumatic grief. PLoS One.

[12] Parisi A, Sharma A, Howard MO, Blank Wilson A (2019). The relationship between substance misuse and complicated grief: a systematic review. J Subst Abuse Treat.

[13] Peinado V, Valiente C, Contreras A, Trucharte A, Butter S, Murphy J, Shevlin M (2024). ICD-11 prolonged grief disorder: prevalence, predictors, and co-occurrence in a large representative sample. Int J Psychol.

[14] Pérez HC, Ikram MA, Direk N, Tiemeier H (2018). Prolonged grief and cognitive decline: a prospective population-based study in middle-aged and older persons. Am J Geriatr Psychiatry.

[15] Croxall J, Foster L, Woodthorpe K (2016). Bereavement support in later life: an emerging social problem for the twenty-first century. Death and Social Policy in Challenging Times.

[16] Lundorff M, Holmgren H, Zachariae R, Farver-Vestergaard I, O'Connor M (2017). Prevalence of prolonged grief disorder in adult bereavement: a systematic review and meta-analysis. J Affect Disord.

[17] Horackova K, Kopecek M, Machů V, Kagstrom A, Aarsland D, Motlova LB, Cermakova P (2019). Prevalence of late-life depression and gap in mental health service use across European regions. Eur Psychiatry.

[18] Luck-Sikorski C, Stein J, Heilmann K, Maier W, Kaduszkiewicz H, Scherer M, Weyerer S, Werle J, Wiese B, Moor L, Bock JO, König HH, Riedel-Heller SG (2017). Treatment preferences for depression in the elderly. Int Psychogeriatr.

[19] (2016). Ältere Menschen in Deutschland und der EU. Statistisches Bundesamt.

[20] Kundt K (2017). Die Gesundheitsversorgung in Deutschland aus staatlicher Sicht: Der Weg zu mehr Vergleichbarkeit und Qualität im System der gesetzlichen Krankenversicherung. Deutsches Institut für Urbanistik.

[21] Andersson G, Cuijpers P, Carlbring P, Riper H, Hedman E (2014). Guided internet-based vs. face-to-face cognitive behavior therapy for psychiatric and somatic disorders: a systematic review and meta-analysis. World Psychiatry.

[22] Arnberg FK, Linton SJ, Hultcrantz M, Heintz E, Jonsson U (2014). Internet-delivered psychological treatments for mood and anxiety disorders: a systematic review of their efficacy, safety, and cost-effectiveness. PLoS One.

[23] Stein J, Röhr S, Luck T, Löbner M, Riedel-Heller SG (2018). [Indication and evidence of internationally developed online coaches as intervention for mental illness - a meta-review]. Psychiatr Prax.

[24] Reck J, Gawlytta R, Kesselmeier M, Böttche M, Niemeyer H, Knaevelsrud C, Rosendahl J (2023). [Differential effects of an internet-based cognitive-behavioral writing therapy for reducing PTSD symptoms after intensive care: results of a per-protocol analysis]. Psychiatr Prax.

[25] Dominick SA, Irvine AB, Beauchamp N, Seeley JR, Nolen-Hoeksema S, Doka KJ, Bonanno GA (2009). An internet tool to normalize grief. Omega (Westport).

[26] Eisma MC, Boelen PA, van den Bout J, Stroebe W, Schut HA, Lancee J, Stroebe MS (2015). Internet-based exposure and behavioral activation for complicated grief and rumination: a randomized controlled trial. Behav Ther.

[27] Kersting A, Dölemeyer R, Steinig J, Walter F, Kroker K, Baust K, Wagner B (2013). Brief internet-based intervention reduces posttraumatic stress and prolonged grief in parents after the loss of a child during pregnancy: a randomized controlled trial. Psychother Psychosom.

[28] Brodbeck J, Berger T, Biesold N, Rockstroh F, Znoj HJ (2019). Evaluation of a guided internet-based self-help intervention for older adults after spousal bereavement or separation/divorce: a randomised controlled trial. J Affect Disord.

[29] Litz BT, Schorr Y, Delaney E, Au T, Papa A, Fox AB, Morris S, Nickerson A, Block S, Prigerson HG (2014). A randomized controlled trial of an internet-based therapist-assisted indicated preventive intervention for prolonged grief disorder. Behav Res Ther.

[30] Schladitz K, Förster F, Löbner M, Welzel F, Stein J, Luppa M, Riedel-Heller SG (2020). [Grief and loss in elderly people: a qualitative study regarding the user acceptance of an internet-based self-help program from user and expert perspective]. Z Evid Fortbild Qual Gesundhwes.

[31] Karyotaki E, Efthimiou O, Miguel C, Bermpohl FM, Furukawa TA, Cuijpers P, Riper H, Patel V, Mira A, Gemmil AW, Yeung AS, Lange A, Williams AD, Mackinnon A, Geraedts A, van Straten A, Meyer B, Björkelund C, Knaevelsrud C, Beevers CG, Botella C, Strunk DR, Mohr DC, Ebert DD, Kessler D, Richards D, Littlewood E, Forsell E, Feng F, Wang F, Andersson G, Hadjistavropoulos H, Christensen H, Ezawa ID, Choi I, Rosso IM, Klein JP, Shumake J, Garcia-Campayo J, Milgrom J, Smith J, Montero-Marin J, Newby JM, Bretón-López J, Schneider J, Vernmark K, Bücker L, Sheeber LB, Warmerdam L, Farrer L, Heinrich M, Huibers MJ, Kivi M, Kraepelien M, Forand NR, Pugh N, Lindefors N, Lintvedt O, Zagorscak P, Carlbring P, Phillips R, Johansson R, Kessler RC, Brabyn S, Perini S, Rauch SL, Gilbody S, Moritz S, Berger T, Pop V, Kaldo V, Spek V, Forsell Y, Individual Patient Data Meta-Analyses for Depression (IPDMA-DE) Collaboration (2021). Internet-based cognitive behavioral therapy for depression: a systematic review and individual patient data network meta-analysis. JAMA Psychiatry.

[32] Te Braake E, Vaseur R, Grünloh C, Tabak M (2025). The state of the art of eHealth self-management interventions for people with chronic obstructive pulmonary disease: scoping review. J Med Internet Res.

[33] Pabst A, Löbner M, Stein J, Luppa M, Kersting A, König HH, Riedel-Heller SG (2020). Internet-based cognitive behavior therapy only for the young? A secondary analysis of a randomized controlled trial of depression treatment. Front Psychiatry.

[34] Welzel FD, Löbner M, Quittschalle J, Pabst A, Luppa M, Stein J, Riedel-Heller SG (2021). Loss and bereavement in late life (60+): study protocol for a randomized controlled trial regarding an internet-based self-help intervention. Internet Interv.

[35] Eysenbach G, CONSORT-EHEALTH Group (2011). CONSORT-EHEALTH: improving and standardizing evaluation reports of web-based and mobile health interventions. J Med Internet Res.

[36] World Medical Association (2013). World Medical Association Declaration of Helsinki: ethical principles for medical research involving human subjects. JAMA.

[37] Shear KM, Jackson CT, Essock SM, Donahue SA, Felton CJ (2006). Screening for complicated grief among Project Liberty service recipients 18 months after September 11, 2001. Psychiatr Serv.

[38] Beck AT, Steer RA, Brown GK (1996). Manual for the Beck Depression Inventory-II.

[39] Hautzinger M, Keller F, Kühner C (2006). BDI-II. Beck Depressions Inventar Revision—Manual.

[40] Prigerson HG, Horowitz MJ, Jacobs SC, Parkes CM, Aslan M, Goodkin K, Raphael B, Marwit SJ, Wortman C, Neimeyer RA, Bonanno GA, Block SD, Kissane D, Boelen P, Maercker A, Litz BT, Johnson JG, First MB, Maciejewski PK (2009). Prolonged grief disorder: psychometric validation of criteria proposed for DSM-V and ICD-11. PLoS Med.

[41] Pfoh G, Rosner R, Rosner R, Pfoh G, Rojas R, Brandstätter M, Rossi R, Lumbeck G, Kotoucova M, Hagl M, Geissner E (2015). Deutsche überarbeitete Übersetzung des PG-13: Erhebungsbogen für anhaltende Trauer. Anhaltende Trauerstörung: Manuale für die Einzel- und Gruppentherapie.

[42] Rosner R, Pfoh G, Rojas R, Brandstätter M, Rossi R, Lumbeck G, Kotoucová M, Hagl M, Geissner E (2015). Anhaltende Trauerstörung: Manuale für die Einzel- und Gruppentherapie.

[43] Wittkowski J (2013). Würzburger Trauerinventar (WüTi). Mehrdimensionale Erfassung des Verlusterlebens.

[44] Bosc M, Dubini A, Polin V (1997). Development and validation of a social functioning scale, the Social Adaptation Self-evaluation Scale. Eur Neuropsychopharmacol.

[45] Duschek S, Schandry R, Hege B (2003). Soziale Aktivität Selbstbeurteilungs-Skala (SASS): Diagnostik Sozialer Funktionsstörungen bei Depressiven Störungen.

[46] Lubben J, Blozik E, Gillmann G, Iliffe S, von Renteln Kruse W, Beck JC, Stuck AE (2006). Performance of an abbreviated version of the Lubben Social Network Scale among three European community-dwelling older adult populations. Gerontologist.

[47] Ware J Jr, Kosinski M, Keller SD (1996). A 12-Item Short-Form Health Survey: construction of scales and preliminary tests of reliability and validity. Med Care.

[48] De Jong Gierveld J, Tilburg T (2006). A 6-item scale for overall, emotional, and social loneliness. Res Aging.

[49] Kilian R, Becker T, Schleuning G, Welschehold M, Hertle C, Hörand S, Matschinger H (2012). Die Entwicklung eines standardisierten Verfahrens zur Messung von Empowerment im Prozess der psychiatrischen Behandlung von Patienten mit schweren psychischen Erkrankungen. YUMPU.

[50] Brooke J, Jordan PW, Thomas B, McClelland IL, Weerdmeester B (1996). SUS: a 'quick and dirty' usability scale. Usability Evaluation in Industry.

[51] Rammstedt B, John OP (2007). Measuring personality in one minute or less: a 10-item short version of the Big Five Inventory in English and German. J Res Pers.

[52] Boelen PA, de Keijser J, van den Hout MA, van den Bout J (2007). Treatment of complicated grief: a comparison between cognitive-behavioral therapy and supportive counseling. J Consult Clin Psychol.

[53] Löbner M, Pabst A, Stein J, Dorow M, Matschinger H, Luppa M, Maroß A, Kersting A, König HH, Riedel-Heller SG (2018). Computerized cognitive behavior therapy for patients with mild to moderately severe depression in primary care: a pragmatic cluster randomized controlled trial (@ktiv). J Affect Disord.

[54] Röhr S, Jung FU, Pabst A, Grochtdreis T, Dams J, Nagl M, Renner A, Hoffmann R, König HH, Kersting A, Riedel-Heller SG (2021). A self-help app for Syrian refugees with posttraumatic stress (Sanadak): randomized controlled trial. JMIR Mhealth Uhealth.

[55] Pohlkamp L, Kreicbergs U, Prigerson HG, Sveen J (2018). Psychometric properties of the Prolonged Grief Disorder-13 (PG-13) in bereaved Swedish parents. Psychiatry Res.

[56] Dominguez-Rodriguez A, Sanz-Gomez S, González Ramírez LP, Herdoiza-Arroyo PE, Trevino Garcia LE, de la Rosa-Gómez A, González-Cantero JO, Macias-Aguinaga V, Miaja M (2023). The efficacy and usability of an unguided web-based grief intervention for adults who lost a loved one during the COVID-19 pandemic: randomized controlled trial. J Med Internet Res.

[57] Wagner B, Rosenberg N, Hofmann L, Maass U (2020). Web-based bereavement care: a systematic review and meta-analysis. Front Psychiatry.

[58] Zuelke AE, Luppa M, Löbner M, Pabst A, Schlapke C, Stein J, Riedel-Heller SG (2021). Effectiveness and feasibility of internet-based interventions for grief after bereavement: systematic review and meta-analysis. JMIR Ment Health.

[59] Faltinsen E, Todorovac A, Staxen Bruun L, Hróbjartsson A, Gluud C, Kongerslev MT, Simonsen E, Storebø OJ (2022). Control interventions in randomised trials among people with mental health disorders. Cochrane Database Syst Rev.

[60] Cristea IA (2019). The waiting list is an inadequate benchmark for estimating the effectiveness of psychotherapy for depression. Epidemiol Psychiatr Sci.

[61] Tur C, Campos D, Suso-Ribera C, Kazlauskas E, Castilla D, Zaragoza I, García-Palacios A, Quero S (2022). An internet-delivered cognitive-behavioral therapy (iCBT) for prolonged grief disorder (PGD) in adults: a multiple-baseline single-case experimental design study. Internet Interv.

[62] Brog NA, Hegy JK, Berger T, Znoj H (2022). Effects of an internet-based self-help intervention for psychological distress due to COVID-19: results of a randomized controlled trial. Internet Interv.

[63] Scott H, Sivell S, Longo M, Seddon K, Fitzgibbon J, Nelson A, Byrne A, Harrop E (2022). What should good bereavement service support look like? Findings from pre-pandemic workshop discussions interpreted in the context of the Covid-19 pandemic. Bereavement.

[64] Nikelski A, Trompetter EM, Boekholt M, Schumacher-Schönert F, Rädke A, Michalowsky B, Vollmar HC, Hoffmann W, Driessen M, Thyrian JR, Kreisel SH (2024). [Everyday life and mental health of elderly with cognitive impairment during the Covid-19 pandemic]. Psychiatr Prax.

[65] Harrop E, Goss S, Farnell D, Longo M, Byrne A, Barawi K, Torrens-Burton A, Nelson A, Seddon K, Machin L, Sutton E, Roulston A, Finucane A, Penny A, Smith KV, Sivell S, Selman LE (2021). Support needs and barriers to accessing support: baseline results of a mixed-methods national survey of people bereaved during the COVID-19 pandemic. Palliat Med.

[66] Goveas JS, Shear MK (2020). Grief and the COVID-19 pandemic in older adults. Am J Geriatr Psychiatry.

[67] Kaiser J, Nagl M, Hoffmann R, Linde K, Kersting A (2022). Therapist-assisted web-based intervention for prolonged grief disorder after cancer bereavement: randomized controlled trial. JMIR Ment Health.

[68] D'Adamo L, Paraboschi L, Grammer AC, Fennig M, Graham AK, Yaeger LH, Newman MG, Wilfley DE, Taylor CB, Eisenberg D, Fitzsimmons-Craft EE (2023). Reach and uptake of digital mental health interventions based on cognitive-behavioral therapy for college students: a systematic review. J Behav Cogn Ther.

[69] Leung C, Pei J, Hudec K, Shams F, Munthali R, Vigo D (2022). The effects of nonclinician guidance on effectiveness and process outcomes in digital mental health interventions: systematic review and meta-analysis. J Med Internet Res.

[70] Richards D, Richardson T (2012). Computer-based psychological treatments for depression: a systematic review and meta-analysis. Clin Psychol Rev.

[71] Carr D, Bodnar-Deren S, Uhlenberg P (2009). Gender, aging and widowhood. International Handbook of Population Aging.

[72] Mackenzie CS, Gekoski WL, Knox VJ (2006). Age, gender, and the underutilization of mental health services: the influence of help-seeking attitudes. Aging Ment Health.

[73] Hardy SE, Allore H, Studenski SA (2009). Missing data: a special challenge in aging research. J Am Geriatr Soc.

